# Making a success of providing NHS Health Checks in community pharmacies across the Tees Valley: a qualitative study

**DOI:** 10.1186/1472-6963-11-222

**Published:** 2011-09-19

**Authors:** Rebekah J McNaughton, Nigel TA Oswald, Janet S Shucksmith, Peter J Heywood, Pat S Watson

**Affiliations:** 1 Health and Social Care Institute, School of Health and Social Care, Teesside University, Middlesbrough, Tees Valley, UK; 2 (formerly) NHS Middlesbrough, Riverside House, 18 High Force Road, Riverside Park, Middlesbrough, Tees Valley, UK

## Abstract

**Background:**

In England and Wales, the Department of Health introduced a primary prevention programme, NHS Health Checks, to provide screening for cardiovascular risk amongst people aged 40-74. The aim of this programme is to offer treatment and advice to those identified with an increased risk of cardiovascular diseases (CVD).

The North East of England has some of the highest rates of CVD in the UK and prevention is therefore a priority. NHS Tees funded this programme of work under the local branding of Healthy Heart Checks (HHC). These were initially implemented principally through GP practices from October 2008 but, in order to mitigate the possibility that some hard to reach communities would be reluctant to engage with some primary care settings, plans were also developed to deliver the programme through workplace settings and through community pharmacies. This paper reports specifically on the findings from the evaluation in respect of the setting up of HHCs in community pharmacies and aims to offer some lessons for other service settings where this option is seen as a way of providing low threshold services which will minimise inequalities in intervention uptake.

**Methods:**

In assessing the community pharmacy component of HHCs, a selection of staff having direct involvement in the process was invited to take part in the evaluation. Interviews were carried out with representatives from community pharmacy, staff members from the commissioning Primary Care Trusts and with Local Pharmaceutical Committee members.

**Results:**

Evaluation and analysis identified challenges which should be anticipated and addressed in initiating HHC in community pharmacies. These have been categorised into four main themes for discussion in this paper: (1) establishing and maintaining pharmacy Healthy Heart Checks, (2) overcoming IT barriers, (3) developing confident, competent staff and (4) ensuring volume and through flow in pharmacy.

**Conclusions:**

Delivering NHS health checks through community pharmacies can be a complex process, requiring meticulous planning, and may incur higher than expected costs. Findings from our evaluation provide insight into possible barriers to setting up services in pharmacies which may help other commissioning bodies when considering community pharmacy as a location for primary prevention interventions in future.

## Background

The most common cause of death and premature disability worldwide is cardiovascular disease (CVD) [1,2], and in the UK diseases of this type cause high level of chronic illness which affect the quality of life of many and result in costly demands in terms of providing health services at every level. The impact of the disease is disproportionately felt amongst more disadvantaged populations, not least because modifiable risk factors such as diet, smoking and physical activity significantly contribute to the prevalence of CVD [3].

Reducing this illness burden has therefore become a major goal of the UK government, and in England and Wales, the Department of Health has introduced a national programme to screen individuals aged 40-74 for early warning signs of cardiovascular risk. This programme was branded as NHS Health Checks (NHS HC). The aim of the programme is to reduce premature morbidity and mortality associated with CVD [3]. In the Tees Valley Healthy Heart Checks (HHC) were established ahead of the national guidance, in response to recommendations made locally by the Health Inequalities National Support Team.

There is ample evidence, however, that such well meant, population-level interventions may actually contribute to widening health inequalities [4]. This intervention, for instance, is primarily offered through GP practices, but studies have shown that some groups of the public rarely visit their GP [5] and it was therefore a prime aim of the HHC programme to avoid making the inequalities worse by excluding groups which may be more vulnerable and at risk anyway. From the start of planning the programme in Tees (April 2008), easy access to checks in workplaces and community pharmacy settings was seen as a valuable addition to assessments offered in general practitioner surgeries.

This paper reports on the phase of initiation of the programme through community pharmacies as part of the strategic approach to deliver HHCs in a variety of community settings.

Delivery of vascular risk assessments in community pharmacy has been widely encouraged as a means of reaching out to people who may be more reluctant to access screening services at other venues [6,7]. In recent years the face of community pharmacy has shifted from one characterised predominantly by small single-handed pharmacies that dealt mainly with issuing prescribed medications, to one which has seen the rise of the multiple chain pharmacy which can offer many additional services [8,9]. It has therefore been argued that pharmacy is now ideally placed to offer public health interventions, screening services and to deliver basic lifestyle advice in the hope of widening access to health services [8,10-12]. In a large scale survey of the general public, Krska and Morecroft [13] report that the general public tend to access pharmacy for specific reasons, primarily to have prescriptions dispensed and to purchase medicines and non medical items. They use the service less frequently to seek out health advice A market research survey carried out by Readers Digest [14] indicate that people in relatively more deprived social classes, however, use community pharmacies more frequently than their affluent counterparts for health information and advice. In part this may be attributed to longer opening hours and access without appointment, but possibly also because pharmacies are regarded as less dauntingly professional in their response to clients [6].

Despite the government's endorsement for this approach, a recent review of the changing role of community pharmacy in the NHS noted the limited evidence for effectiveness and value for money of services such as screening in pharmacies [9]. Patel [15] also cautioned about the use of non NHS providers to deliver CVD assessments, and called for the development of robust data systems that could handle the transfer of data between the non NHS provider and primary care providers without duplicating results.

In a small scale, real world trial delivering targeted vascular assessments through community pharmacies in Birmingham, Horgan et al showed that they had achieved particular success in engaging individuals from minority ethnic groups, [10] suggesting that pharmacy may indeed be useful in reducing some health inequalities. Horgan et al went on to report that men - a segment of the population renowned for not attending services in GP practices [16] - had engaged with the programme delivered through community pharmacy [6]. This suggests that in the real world pharmacy may offer a better chance of engaging certain segments of the population. However, there is conflicting evidence which suggests that the general public do not always view pharmacy as a place to receive lifestyle advice or screening, owing to concerns about lack of capacity amongst pharmacy staff and confidentiality [13]. It is thus far from clear whether community pharmacy offers the panacea 'low threshold' service that commissioners had hoped for, in the sense of providing easy access services at times to suit the patient, without an appointment and provided by staff who are intrinsically more accessible and approachable.

The evaluation of the initiation of community pharmacy based screening in the Tees Valley allows us to share insights which may help other commissioning bodies both to assess realistically what is involved in setting up such assessments, and to model appropriately the likely cost per assessment. The complexity of population primary prevention and the emphasis on this as a future means of achieving health gain without increasing inequality suggests that our findings may provide lessons beyond the system we studied.

## Methods

The study described in this paper was part of an overall evaluation which used a Theory of Change [17] framework, entailing a process of continual feedback between the researchers and the organisations being evaluated to provide a clear model of the logic of the programme, to shape the direction of programme change and provide evidence for suggested modifications. In this respect the commissioning organisation was provided with constant updates about findings from the evaluation. Staff in the delivery and implementation arms of the intervention kept the research team abreast of all changes made to the initial programme protocol.

### Participants

Ten Primary Care Trust (PCT) members of staff who had direct professional involvement in setting up HHCs in community pharmacy were invited to take part in interviews. Staff included the Director of Public Health, project manager, clinical lead, public health nurses, pharmacy advisor (medicines management), community services manager, a professional executive committee member and the IT database developer.

All nine pharmacies that were hoping to take part in the pilot phase of the HHC programme were contacted and invited to take part in the evaluation. Eight pharmacists agreed to be interviewed. Pharmacy customers were not included within this evaluation.

Two representatives from the Local Pharmaceutical Committee (LPC) also took part in the evaluation.

### Data collection

Each participant was contacted by letter and provided with written information about the aims of the project and what would be required of them should they agree to participate.

One-to-one interviews were undertaken with each participant. These interviews followed a semi structured format and centred around the setting up of the HHC programme within pharmacies. PCT members of staff and pharmacists were interviewed about the selection and recruitment of pharmacies into the HHC programme; clinical governance and health and safety issues; development of the Service Level Agreement (SLA); pharmacy accommodation; training provision and IT development, data collection and transfer. LPC members were asked about the role of the LPC in developing the SLA. Anonymity and confidentiality were assured and each participant was encouraged to be frank and open about their experiences of setting up the HHCs in community pharmacy. Each interview lasted between 20 and 60 minutes and was, with the consent of the participant, digitally recorded. Interviews were subsequently transcribed verbatim. All names, places of work and other identifying features were removed from the text of the transcriptions to avoid participants being identified. Pharmacists were interviewed at two time points, once before and once after they went live delivering the HHC assessments.

### Data analysis

Transcriptions of each interview were read and independently scrutinised by two members of the research team. Data were then analysed and coded using a thematic content analysis [18] framework. Each analyst read all transcripts several times to familiarise him/herself with the issues raised and developed a coding framework to establish themes. Initial codes were identified independently and all data supporting the codes were highlighted in the transcripts. These initial codes were then grouped together to form themes which were then corroborated between analysts. Once agreement had been achieved between analysts, these themes were written into a narrative form to provide an accurate illustration of the theme using quotations taken directly from the transcripts.

The School of Health and Social Care Ethics Committee at Teesside University scrutinised and approved this study protocol.

## Results

Eight pharmacies signed up to the Service Level Agreement and took part in the pilot phase of HHC delivery across the Tees Valley. Of these eight pharmacies six were small pharmacist led businesses that were located out in community settings and two were large multiples one of which was situated in a supermarket chain the other in a purpose built 'health village'. Pharmacies were required to provide CVD risk assessment to individuals, calculate possible CVD risk over the next ten years and signpost to medical intervention or offer lifestyle advice as appropriate. In this section we provide an overview of findings from the evaluation, grouped together under the main emergent themes; establishing and managing pharmacy Healthy Heart Checks, overcoming IT barriers, developing confident, competent staff, ensuring volume and through flow in pharmacy.

### Establishing and managing pharmacy Healthy Heart Checks

It was decided in the Tees Valley that HHCs would be delivered through a selection of community pharmacies as a method to engage hard to reach populations that would not necessarily access this type of service through a GP practice. Guidance from the Department of Health suggested that PCTs look outside of GP practices as sites to deliver the NHS HC programme and that they should consider community based venues. PCT staff felt, at the time, there was sufficient evidence from around the UK to support the inclusion of community pharmacy as a place to offer the HHC programme.

*The Department of Health had said they wanted us to look at other providers [other than GP practices], and community pharmacies, they thought, would be a good venue. There had been projects around the country like the big one in Birmingham that was very successful, fantastic results, doing CVD assessments*. (PCT staff member)

It was assumed that pharmacies had become trusted sources of information within the community, through delivering services such as smoking cessation. Pharmacies also have a wide customer group which tends to access their services on a regular basis, again making them potentially ideal locations to situate a service like the HHC programme and providing a low threshold, non threatening service that might be less likely to increase health inequalities:

*From my experience of working in a deprived area, as a health visitor, people do access pharmacies like they would access their GPs at times. They trust their pharmacy, and from being in [names pharmacy] that is very evident. It's 'Hi Tom. I need this, I need that. What can I do about this? Can I try that?' They [the pharmacists] know their population very well*. (PCT staff member)

Accordingly, a plan was made to involve community pharmacies as a major partner in cardiovascular risk assessment, and initial preparations were made for marketing to the public the availability of checks in pharmacies. It was largely taken for granted that the initiation of checks in willing pharmacies would be straightforward and quick. In the event, however, implementation proved to be very complex, and the initiation of assessments (even in a small pilot group of pharmacies) was delayed by over a year.

Pharmacists were eager at first to be involved in delivering extra services, as the HHCs were potentially both a method of generating extra revenue and bringing more people into their stores:

*We do focus on the services, we do a lot of morning after pills. We have started doing the flu and cervical cancer vaccinations. So in this store we like doing the services, because it brings people into the store *(Pharmacy 1 representative)

As the Tees service began to roll forward, information began to be fed back, through the national NHS HC network, from other areas in the country that had been delivering checks through pharmacies. Some commissioners had begun to withdraw services from pharmacies because low levels of uptake had made them non viable.

*[In other PCT areas] it has not worked for them. For instance in [names area], they had 30 pharmacies set up and they have taken the equipment off half of them because there was no activity*. (PCT staff member)

During the initial set up for delivering HHCs through the Tees pharmacies many changes took place within the PCT. Swine flu hit the UK and required rapid and immediate response. Staff who had been involved with the early stages of managing the HHC programme were not able to see it through to completion, which also impacted on the HHC programme.

The PCT aimed to recruit pharmacies against set inclusion criteria that related to making sure pharmacies played a major part in addressing issues of disadvantage. However, after recruitment of pharmacies began, it soon became apparent that not all pharmacy accommodation was of a sufficient standard to deliver the HHCs. Each pharmacy was required to have in place a private consultation room with secure access to the Internet and hand washing facilities, but there was huge variability between pharmacies in the appropriateness of the accommodation:

*Some [spaces offered for checks], are brilliant, whereas some were like an old broom cupboard. I would have liked to have visited them myself beforehand *(PCT staff member)

Much work went into developing a Service Level Agreement for the HHC, this took over a year to develop. Many obstacles became apparent during this time, which had not been anticipated. It became clear that payments needed to be handled differently to those for GP practices, as pharmacies pay VAT on all services they deliver. This took time to resolve and required much input from organisations outside of the PCT:

*We had to go away and create prices that would actually work; a set up fee, an annual fee and then a small fee for actually doing the service *(LPC member)

Additional resources had to be assigned to the pharmacy roll out of the programme when it became apparent that, unlike staff working in other settings such as GP practices, pharmacy staff did not have the relevant vaccinations to enable them to deal with blood and bodily fluids. To this end all staff delivering the HHCs had to receive Hepatitis B vaccinations, which had an additional impact on the budget as clarified by a PCT staff member:

*We have actually paid a lot of the pharmacies to have Hepatitis B vaccinations which is a requirement for nurses and GPs who deal with bodily fluids. We have actually paid for that out of our CVD budget *(PCT staff member)

### Overcoming IT barriers

It became clear very quickly that pharmacies did not have a sufficiently secure Internet connection to allow them to transfer patient identifiable data to the NHS server which held the HHC database. To overcome this problem pharmacies were required to upgrade their Internet connection and were issued with a special 'RAS' token for extra security. This RAS token generated random number combinations which increased security when accessing the NHS server, it worked in a similar way to a card reader for Internet banking. The requirement for increased Internet security and the use of such tokens posed much more of a problem for some of the larger chain pharmacies than the smaller independent ones. Larger chains found that their company policies dictated that they could not have open access to the Internet, which was one of the requirements to enable connection to the PCT server. For this reason some of the larger chain pharmacies were unable to take part in this roll out of HHCs in pharmacy. For those pharmacies that were able to connect freely to the Internet and met all of the security requirements, they were able to trial the database they would be using, and felt that it was user friendly:

*It is nice that the computer system leads you through step-by-step *(Pharmacy 7 representative)

Once the system went live however, it was evident that there were some technical errors which affected the calculation of patients' risk scores. This was attended to promptly by the PCT: however it did affect pharmacists' confidence in the system.

### Developing confident, competent staff

From the PCT's point of view the training needs of pharmacy staff were much greater than initially envisaged. It had originally been expected that pharmacists would deliver the service to customers themselves, but it soon became clear that this was impractical due to the fact that many pharmacists are sole traders and have a variety of other daily commitments. It was then agreed that pharmacy assistants could carry out the initial assessment which involved; taking anthropometric measurements from the patients, medical history, blood pressure and blood samples. This meant that the pharmacist would only be involved at the end of the consultation, delivering the final risk score and giving lifestyle advice to the patient. This increased the training load significantly in order to make sure that pharmacy assistants were competent to carry out the assessment:

*The pharmacists are highly skilled and highly trained. The people they have working for them, who are normally dispensing assistants, haven't got the background in care knowledge or expertise. It wasn't like a GP surgery where you have Healthcare Assistants and Practice Nurses who on a day to day basis take blood pressures, take pulses, take blood and give advice on health *(PCT staff member)

To address this issue a training package was developed and delivered by the PCTs and it was well received by pharmacy staff. Due to the nature of pharmacy businesses it soon became apparent that training would need to take place out of office hours. The PCT took a flexible approach to this, which was appreciated by pharmacy staff as their needs had been taken into consideration. There was however quite a time lag between pharmacy staff being trained and then beginning to deliver the service to members of the public, and this then necessitated further refresher training from the PCT. Once trained, pharmacy assistants felt that delivering the HHC service was a good opportunity for staff development and were pleased with the increased responsibility that went with it:

*One of our counter staff...said 'I never thought I would be doing this!' She's quite excited and it's a huge jump from their present role *(PCT staff member).

In pharmacies, the HHC programme 'went live' in January 2010. As part of the agreement between PCT and pharmacy, all pharmacy staff were required to be trained and signed off, by the PCT, as competent to deliver the assessments. At the end of the service evaluation (August 2010), not all staff had achieved this. This was due to two main reasons. Uptake of the screening opportunity by the public had been very low, meaning the pharmacy staff had not had the opportunity to deliver many assessments. Pharmacy staff had also required a lot more training than initially anticipated and, even after being given this support, lacked confidence in delivering the new service:

*One girl has already been signed off. The other lady is just waiting to be signed off. She is still a bit nervous about getting bloods from people *(Pharmacy 6 representative)

### Ensuring volume and through flow in pharmacy

Across the seven pharmacies that began to deliver the HHC programme, uptake has been low. Between January and September 2010, 204 assessments had taken place in pharmacies across the Tees Valley. During the same period, 2082 assessments had been completed in workplaces and 16,050 in GP practices across Tees. The number of completed assessments varied greatly from pharmacy to pharmacy, ranging from 9 assessments in one pharmacy to 49 in another. All pharmacies felt disappointed with this level of uptake, despite the PCT advertising the service within each pharmacy and through adverts in the local press. Pharmacy staff also advertised the service to patients entering their stores to try and raise awareness and increase uptake of the service themselves but with very little effect. Pharmacists tried to give reasons why uptake had been low despite efforts to raise awareness of the service they were offering. Some felt that it may have been due to their geographic locations and low foot-fall in store:

*I don't know if it's our location, that there isn't enough people coming through *(Pharmacy 4 representative)

This view was also held by some PCT staff, who added that some of the pharmacists delivering HHCs were very close to GP practices that were also delivering the HHC, introducing competition between providers:

*Some [pharmacies] are really struggling because they are out of town and in locations close to GP practices - which is where the pharmacies get a lot of their business from *(PCT staff member)

This view was held by pharmacists too. They felt pharmacies and GP practices were in direct competition, trying to entice a finite number of patients into what was, essentially, the same service:

*Actually there's another problem, capturing the people. Everyone is out to capture them...it's very hard if you see someone coming in and say, 'Oh! You could be a candidate', and they say, 'The surgery has approached me and I'm going there' *(Pharmacy 5 representative)

## Discussion

This study gave us important insights into initiating primary prevention services in community pharmacy and some of the obstacles which, in future, might be avoided. To aid rollout of similar interventions in community pharmacy we would recommend that commissioning bodies critically consider the experience in the Tees Valley. Unforeseen problems resulted in a much delayed service in community pharmacies. The optimistic assumption that it would be simple to carry out HHCs in pharmacies was soon proved wrong. Critical phases in development were too dependent on individuals and contingency plans were not made in the event of sickness or other absence. The priority applied to NHS Health Checks, and the additional work entailed, needed to be made explicit to all of those responsible for operationalising the policy across the PCT directorates.

Because of the complexities involved in setting up this service the true cost per assessment was not anticipated. In advance of implementation the uptake of HHCs in pharmacy could only be estimated, as no intervention of this kind had been delivered through this setting before. Whether the price for the contract should include indirect as well as direct costs and VAT needed to be resolved at an early stage. Ancillary costs, most importantly those for pharmacy staff training, consumables, nursing support and IT support, are an essential part of the calculation and in the case of the Tees PCTs were much higher than originally expected. Moreover, the low throughput of patients could make the set up costs of this type of service uneconomical in the long term.

Commissioners will rightly expect pharmacies delivering HHCs to meet NHS standards for criteria such as privacy of consulting space, availability of hand washing facilities, secure Internet connections for patient data transfer and appropriate application of health and safety procedures, e.g. for disposal of sharps and handling of blood. These criteria are not always met in pharmacies. To establish equity of access for assessments substantial additional investment may be required in pharmacies with poorer facilities to bring them up to expected standards. PCTs need to balance cost, coverage and equity in rolling out pharmacy based health checks.

The SLA that is negotiated with pharmacies must recognise the important differences between general practice and pharmacy provision. In particular, general practice teams, which rely routinely on health care assistants and practice nurses to carry out such procedures, have clinical experience which is not generally mirrored in pharmacies. In a pharmacy team the pharmacist is likely to be the only person with the skills to undertake clinical assessment and communicate the level of risk assessed from the HHC. It is unrealistic to expect the pharmacist him/herself personally to deliver the 30-40 minute health check, especially if an open access policy (i.e. no appointment needed) is being followed. Therefore extra training costs should be considered when considering pharmacy as a venue to deliver this kind of service.

Commissioners need to consider carefully issues such as the VAT payable by pharmacies (but not general practices), and whether costs for the full service (including equipment, disposables, waste disposal and staff immunisation) is included in the detailed service specification.

The question of whether targets for activity should be set is contentious: a minimum level of activity seems necessary to maintain competence and efficient use of disposables, but may be incompatible with the aspiration to make checks available in all pharmacies. The calculation of cost per case, and decisions about value for money, are strongly influenced by both the service specification and HHC activity in individual pharmacies

Many pharmacies do not have the full access to the NHS network which is required for the efficient and secure transfer of information between pharmacy, PCT and general practice, even if a common database and a system of common codes has been agreed. Confidentiality standards and the degree of access permitted to staff within a pharmacy team are important issues.

In establishing HHCs in Tees pharmacies achieving connectivity with the NHS, staff IT training and continuing support proved time consuming and costly. In some cases, the data security practised by national pharmacy chains impeded the loading of software onto pharmacy systems. Once the system was live there were some issues with calculations of risk score which impacted on pharmacists' trust in the system. Thorough piloting of IT systems is essential in these situations.

The extent of training required to bring members of pharmacy teams to the level where they could competently and confidently carry out the HHC process was greatly underestimated. This was made worse by the length of time between initial selection and the activation of the service. Time is required not only for initial training, which is itself a problem given the constraints on releasing both pharmacists and team members during a six-day working week, but there is a need for continuing support to assure clinical governance and quality. The training received by pharmacy teams evaluated very positively, and there was clear evidence that staff felt empowered by the opportunity to add to their role by delivering a clinical service.

## Conclusions

It may be said that all the issues that were identified could have been anticipated. However, being an early adopter of change in a complex intervention entails risks which can be avoided by others as experience accumulates, and our purpose in providing this paper is to indicate areas which deserve early consideration by commissioning bodies. We are aware, from anecdotal evidence, that PCTs in other regions have had similar experiences. We believe that considering the lessons learned in this report will improve the efficiency, increase the timeliness and may well benefit patients if it enhances the effectiveness of the national rollout of NHS Health Checks.

## Competing interests

The authors declare that they have no competing interests. All authors declare that they had (1) no financial support for the submitted work from anyone other than their employer; (2) no financial relationships with commercial entities that might have an interest in the submitted work; (3) no spouses, partners, or children with relationships with commercial entities that might have an interest in the submitted work; (4) no non-financial interests that may be relevant to the submitted work; (5) no personal competing interests.

## Authors' contributions

RM contributed to the design of the project, carried out the fieldwork, analysed the data and was main author of this paper. NO contributed to the design of the project, analysis of data, day to day project management and made significant contributions to the writing of this paper. JS had overall managerial responsibility for the evaluation project, and contributed to the design of the project and the writing of this paper PH and PW contributed to the design of the project and the writing of this paper. PH was responsible for the commissioning of the original intervention and the local evaluation of the scheme. All authors read and approved the final manuscript.

## Pre-publication history

The pre-publication history for this paper can be accessed here:

http://www.biomedcentral.com/1472-6963/11/222/prepub
